# Genomic alterations in mucins across cancers

**DOI:** 10.18632/oncotarget.17934

**Published:** 2017-05-17

**Authors:** Ryan J. King, Fang Yu, Pankaj K. Singh

**Affiliations:** ^1^ The Eppley Institute for Research in Cancer and Allied Diseases, University of Nebraska Medical Center, Omaha, Nebraska, USA; ^2^ Department of Biostatistics, University of Nebraska Medical Center, Omaha, Nebraska, USA; ^3^ Department of Biochemistry and Molecular Biology, University of Nebraska Medical Center, Omaha, Nebraska, USA; ^4^ Department of Pathology and Microbiology, University of Nebraska Medical Center, Omaha, Nebraska, USA; ^5^ Department of Genetics, Cell Biology and Anatomy, University of Nebraska Medical Center, Omaha, Nebraska, USA

**Keywords:** mucins, MUC1, cancer genetics, genetic alterations, cancer genetic profiles

## Abstract

The significance of mucins in cancers has led to the development of novel biomarkers and therapeutic agents against cancers. Despite significant advances in the understanding of mucins, systemic investigations into the role of mucins in cancer biology focusing particularly on the histological subtypes and stages, along with other variables, are yet to be carried out to discover potential novel functions and cancer-specific roles. Here, we investigated 11 mucin expressing cancers for DNA mutations, mRNA expression, copy number, methylation, and the impacts these genomic features may have on patient survival by utilizing The Cancer Genome Atlas dataset. We demonstrate that mucin DNA mutations have a significant rate, pattern, and impact on cancer patient survival depending on the tissue of origin. This includes a frequent T112P mutation in *MUC1* that is seen in half of the pancreatic *MUC1* mutations, as well as being present in other cancers. We also observed a very frequent *MUC4* mutation at H4205, which correlated with survival outcomes in patients. Furthermore, we observed significant alterations in mucin mRNA expression in multiple tumor types. Our results demonstrate *de novo* expression of certain mucins in cancer tissues, including *MUC21* in colorectal cancers. We observed a general decrease in promoter methylation for mucins, which correlated with decreased expression of many genes, such as *MUC15* in kidney cancers. Lastly, several mucin gene loci demonstrated copy number increase in multiple histological subtypes. Thus, our study presents a comprehensive analysis of genomic alterations in mucins and their corresponding roles in cancer progression.

## INTRODUCTION

Mucin-based biomarkers have been utilized in clinic for multiple cancers, highlighting the functional significance of mucins in cancer [1, 2]. Mucins display altered expression and abnormal glycosylation in early and late stages of cancer [3–5]. Multiple membrane-tethered mucins associate with malignant potential and a poor prognosis, while secreted mucins correlate with an improved prognosis [6]. A number of mucin family members have been discovered to possess signaling potential of great significance. MUC1, the most studied mucin that is involved in the pathogenesis of the multiple cancer types, serves as a scaffold, a signaling adaptor, a transcriptional co-activator, and a metabolic and immune regulator [7, 8]. It triggers intracellular signaling, leading to transcriptional changes in the nucleus, in response to alterations in the extracellular microenvironment of the tumor cells [8, 9]. MUC1 intracellular signaling impinges upon a plethora of signaling pathways, including MAPK, NF-kB, JAK-STAT, HIF, Wnt, p53, ERα, and c-Src [4, 10]. Depending on the cellular context and growth cues in extracellular microenvironments, MUC1 also regulates a variety of cellular responses such as growth, differentiation, apoptosis, cell fate, oxidative stress death protection, immunosurveillance, adhesion, polarity, inflammation, colonization, and metabolism [7, 8, 11]. MUC1 expression correlates with poor prognosis [12, 13]. MUC4 is another well-studied mucin that possesses signaling capabilities mainly by allowing increased signaling through ErbB2 [4]. MUC4 expression is associated with proliferation, blocking apoptosis, metastasis, and gemcitabine resistance. Hence, it is no surprise that the increased expression of MUC4 is seen in several types of cancer and associates with poor prognosis [14]. MUC13 is another transmembrane mucin that negatively impacts ovarian cancer patient survival, observed to have roles in increasing cancer cell motility and proliferation [10]. Contrary to these cancer-promoting mucins, MUC2 interacts with inflammatory pathways and helps protect against tumor development [10].

Due to the aberrant expression, signaling regulation and glycosylation of mucins in cancer, mucins have been explored as biomarkers [1, 2, 5, 15]. MUC16 (CA125) is a well-known ovarian cancer marker upregulated in > 80% of cases [4] and serves as a FDA-approved marker for ovarian relapse [3]. It is a possible predictor of prognosis in pancreatic cancer, which also displays an aberrant increase in MUC16 [1, 16]. MUC1 expression is commonly altered; it is seen abnormally expressed in approximately 900,000 of 1.4 million tumors diagnosed in the United States each year [10]. CA19-9 and DU-PAN2 are clinically used markers for MUC1 in pancreatic cancers with the former being FDA approved [3]. N-terminal fragments of MUC1 can be detected in the serum of pancreatic cancer patients by the CA15-3 serum assay, and MUC1 expression together with serum levels are associated with a poor prognosis and recurrence in resected patients [13]. MUC21 may be a good candidate diagnostic biomarker for lung adenocarcinoma [17].

Aside from diagnosis, mucins also serve as markers for aggressive behavior in cancer [10]. In breast cancer, secreted mucins correlate with tumor size, stage, survival, and metastatic potential, while expression of membrane mucins correlate with grade, vascular invasion, metastasis spread, and recurrence [18]. MUC1 is associated with invasion and metastasis in several tumors [6]. MUC3 expression correlates with a poor prognosis and tumor size, invasion, and metastasis [3]. MUC4 associates with poor prognosis in several cancer types and may serve as a potential marker for pancreatic cancer [4, 9, 10, 14]. Contrastingly, high expression of MUC5AC correlates with an increased survival [3], while high MUC1 expression is beneficial in gastric carcinoma prognosis [19]. These studies show the importance of mucins in cancer while highlighting existing differences in cancers that need to be addressed.

Different cancers utilize a variety of mucins that may impact prognosis through multiple mechanisms. These mucins have been utilized as biomarkers and as vaccination targets [3, 14]. However, differences in the roles played by mucin genes have been observed across cancers, with many mucins being understudied. Furthermore, the complete landscape of genomic alterations of mucin has not been studied in many cancers and histological subtypes. Hence, we undertook a pan-mucin genomic study across multiple cancers to investigate potential new avenues and to discover new alterations that may impact the mucin functions in cancers. These tissues include cancers of the breast, bladder, colon, esophagus, kidney, lung, ovary, pancreas, rectum, stomach, and uterine corpus. Furthermore, we explored multiple genomic roles, such as DNA mutations, mRNA expression, copy number, methylation, *de novo* expression and silencing, and examined if these alterations significantly impact survival.

## RESULTS

### Mutation patterns across 11 mucin-expressing tissues

**Figure 1:**
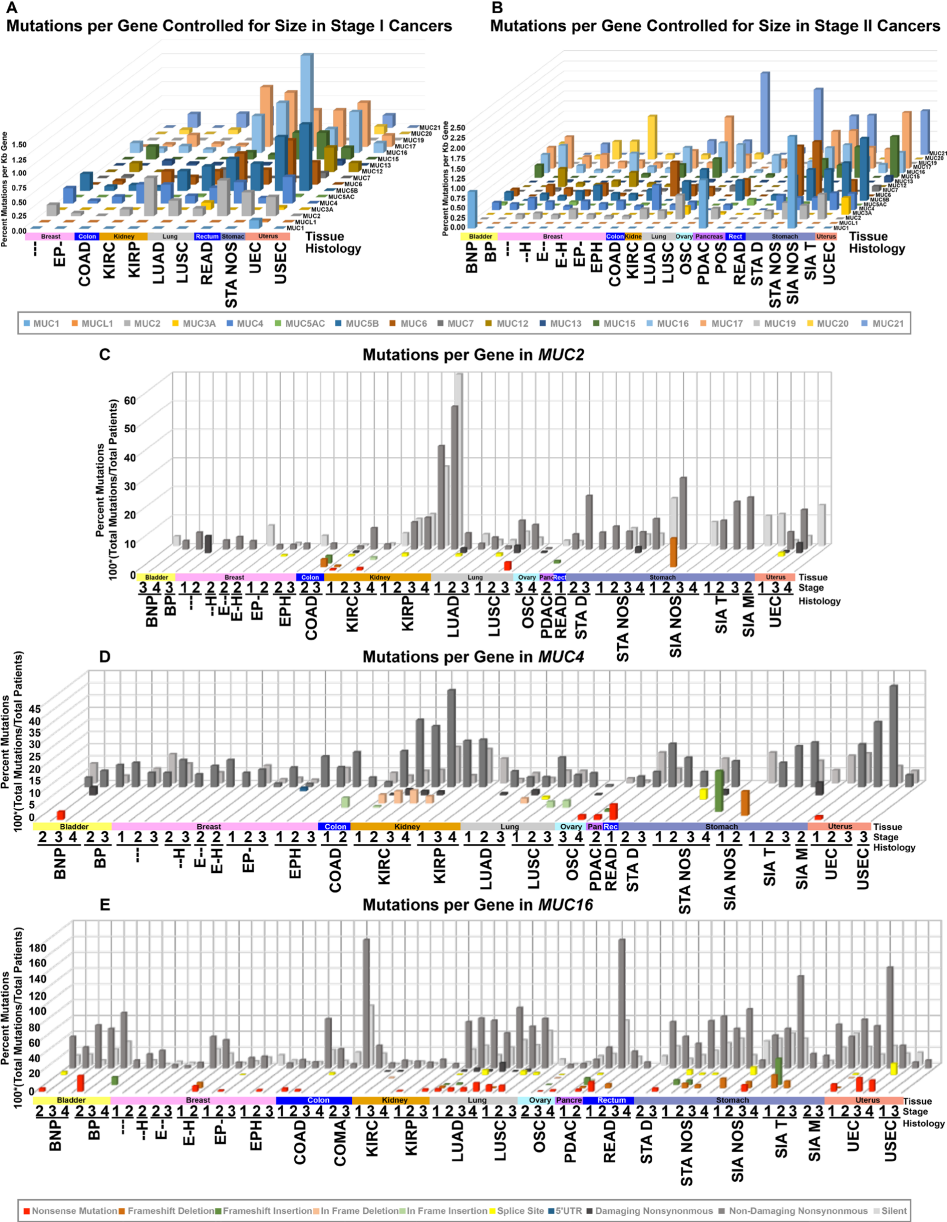
DNA mutations in histology and stage controlled cohorts TCGA mutation data was organized into cohorts based on histology and stage for all patients. Cohort names, stages, and specimen sizes can be found in Supplementary Table 1. Cohorts with 10 or more samples were grouped by stage I (**A**) or stage II (**B**) and had total patients divided by total mutations to achieve percent mutated. This value was normalized to the kilobase pairs (kb) of the longest transcript possible utilizing the transcription start and end coordinates provided by UCSC genome table browser's list of known genes. The specific type of mutation was examined specifically for *MUC2* (**C**), *MUC4* (**D**), and *MUC16* (**E**). The more damaging the mutation category, the closer it appears (C–E). Shades of red indicate deletion, shades of green for gain, yellow for splice site, blue for noncoding, and shades of grey for single nucleotide variations.

Mucins vary considerably in gene size, but when controlling for gene length, distinct mutation rates for individual mucins appear across different tissues and histological cohorts. For normalizing mutations with gene length, we divided the total number of mutations by the number of sequenced patients and then divided by the largest transcript length reported by UCSC from the human genome assembly 19 (hg19) to compare mutation rates relative to size. The rate of mutations varies from mucin to mucin depending on the histological cohort (Figures 1A, 1B, and Supplementary Dataset 1). Full cohort information, acronyms, and sizes can be found in Supplementary Table 1. Kidney papillary cell carcinoma (KIRP) shows the largest relative mutation rate for *MUC2*, while no other histological subtypes appear to favor *MUC2* mutations to a similar degree (Figure 1C). Furthermore, only KIRP shows a strong mutational preference towards *MUC2* and a marginal background mutation rate towards other mucins, suggesting the high mutation rate is not due to a high mutational rate in this cohort. Like KIRP, kidney clear cell carcinoma (KIRC) trends with lower rate of mucin mutations, except for *MUC4*, which shows a cluster of in-frame deletions for KIRC (Figure 1D).

Uterine corpus endometrioid endometrial adenocarcinoma (UEC) appears to acquire more mutations in multiple mucins, including *MUC5B* and *MUC17*, which becomes very prominent in stage III (Figures 1A, 1B, and Supplementary Dataset 1). UEC tumors also have mutations in other mucins, such as *MUC4* and *MUC16*, however, the mutation rates are much lower (Figure 1D and 1E). Uterine serous endometrial adenocarcinoma (USEC) does not appear to show a similar mutational rate for mucins (Supplementary Figure 1). A few notable examples also include *MUC6* and stomach adenocarcinoma not otherwise specified (STA NOS) (Supplementary Dataset 2). Lung squamous cell carcinoma (LUSC) and lung adenocarcinoma (LUAD) share *MUC16* and *MUC17* mutations at a similar rate between the histological subtypes. Lastly, despite many cancers observed not to harbor mutations in *MUC12* and *MUC19*, breast cancer appears to have a unique profile.

### Mutations across mucins

**Figure 2:**
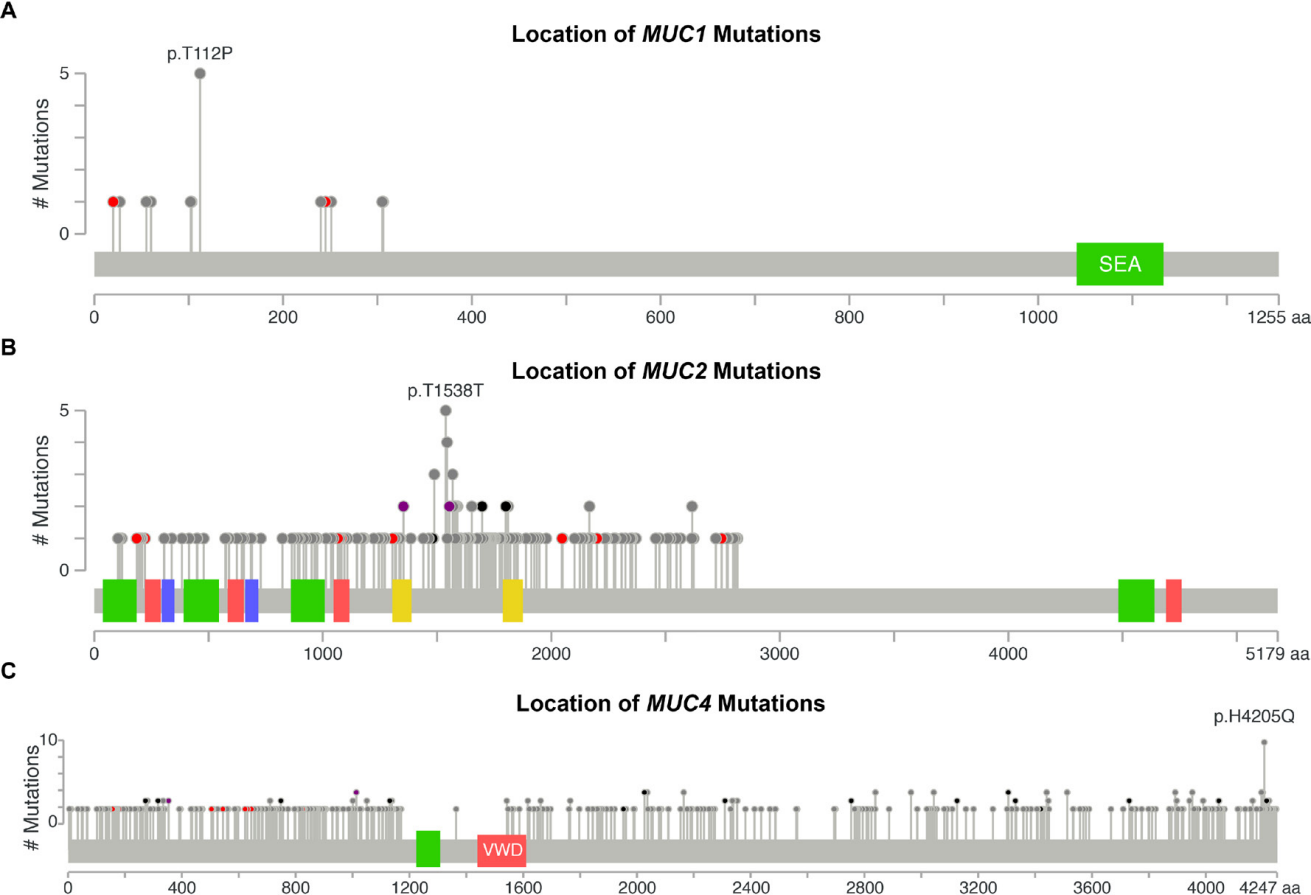
DNA mutations by location DNA mutations in all cohorts were aggregated together to examine for commonly mutated genomic regions. Figures were generated by cBioPortal Mutation Mapper [44, 45], in which each lollipop denotes a unique mutation location for *MUC1* (**A**), *MUC2* (**B**), and *MUC4* (**C**). Exact mutations with patient identifiers can be found in Supplementary Table 1. Red circles indicate a frameshift, nonsense, or a splice site mutation. Black circles denote inframe additions or deletions. Grey circles indicate either silent or nonsynonymous mutations. Purple indicates multiple color categories reside at the same location. Green, red, or yellow bars indicate domains.

Examining the types of mutations and their rates may help decipher the biological significance. Furthermore, location specific mutations may indicate a significant role of the residue or protein domain(s) in cancer pathogenesis. We discovered distinct mutation profiles for mucins, which associated with certain tissue and histological subtypes. A total of five tissues were observed with *MUC1* mutations (Supplementary Table 2). Non-papillary bladder cancer, pancreatic ductal adenocarcinomas (PDAC), and stomach intestinal adenocarcinoma not otherwise specified (SIA NOS) were the only tissues that were observed to have *MUC1* mutation(s) at stage II cancer (Figures 2A and Supplementary Table 2). Half of stage II PDAC *MUC1* mutations are T112P. Altogether, 5/8 of stage II *MUC1* mutations are T112P and 31.25% of *MUC1* mutations observed in all stages were T112P. *MUC2* mutations increase with the increasing disease stage in KIRP, appearing in 9.5% of stage I KIRP (*n* = 95) and up to 50% of stage IV KIRP cancers (*n* = 10). Most of these mutations are non-structurally damaging non-synonymous mutations (Supplementary Dataset 2). *MUC2* shows a large cluster of mutations with a Gaussian distribution across tissues with the mode at T1538 (Figure 2B). Many of the multiple mutations appear to target threonine and appear to include multiple silent mutations, perhaps suggesting a role of the region in regulating transcription, mRNA stability, or non-coding RNAs. Amino acid changes observed in more than one patient in the cluster spanning from residues 1353-1652 target the threonine codon in over 85% of the cases. Within this dense cluster, three non-damaging T1488P mutations are observed in KIRP, while three T1568M mutations are observed once in KIRP, rectal adenocarcinoma (READ), and uterine endometrioid endometrial adenocarcinoma (UEC). UEC has a large percent of the *MUC3A* mutations with 11 out of the 35 of the mutations in *MUC3A*, of which, over a third of the UEC mutations occur at S207, with two in-frame insertions and one non-synonymous mutation (Supplementary Table 2 and Supplementary Dataset 2).

The mutation pattern on *MUC4* suggests that it might play a functional role in KIRC pathogenesis (Figure 2C and Supplementary Table 2). *MUC4* in frame deletions appear in a large fraction (2.9%–5.4%) for KIRC. Furthermore, compared to silent mutations, non-damaging *MUC4* mutations are drastically increased in KIRC, resulting in amino acid changing mutations to be 19.8% in stage I (*n* = 197), 35.0% in stage II (*n* = 40), 32.2% in stage III (*n* = 112), and 44.1% in stage IV (*n* = 68). UEC also has an increased rate of non-damaging mutations for *MUC4* that increase with the increasing stages, ultimately resulting in 41.4% single nucleotide variation (SNVs) mutations in stage III (*n* = 29) (Figure 1D). This dataset reveals all 10 H4205Q MUC4 mutations occur as 10 G < C; half of which are from KIRC, three from bladder cancer, and two from LUAD (Figure 2C and Supplementary Table 2). In KIRC, high rates of in-frame deletions occur compared to other tissues for *MUC4* (Figure 2C). Seven different locations were observed to have an in-frame deletion that was identical to another in-frame deletion observed in another patient (Supplementary Table 2). Only an in-frame insertion of serine at 2026 was seen to match for other tissues (two occurrences in LUSC and once in COAD), but was not observed in KIRC. There are eight locations in which the same resulting amino acid change is observed three times, half of which are only seen in KIRC. Lastly, multiple positions in *MUC4* had at least two mutations at the same position, which overall suggests a role of mutations in *MUC4*, especially in KIRC.

*MUC5B* is another large mucin gene that is mutated the most in UEC with 30.9%, 46.7% and 79.3% amino acid changing mutations in stages I–III (Supplementary Table 2 and Supplementary Dataset 2). Mutations appear evenly spread; however, KIRP shows three D682G mutations, while T4373 shows four deletions, two of which are in-frame that are observed in PDAC, with the remaining in UEC and KIRC (Supplementary Table 2, Supplementary Dataset 2, and Supplementary Figure 1). *MUC6* has a relatively high mutation rate in stage II PDAC, where 9.6% (*n* = 114) of the mutations caused amino acid changes, while no silent mutations were discovered. Furthermore, breast invasive carcinoma (BRCA) harbors three frameshift insertions at L2241, while KIRC has two at P1570. Low frequency of silent mutations were observed with *MUC7*. Three of the seven non-damaging UEC stage I (*n* = 149) mutations were found to be S336L in *MUC7*.

Despite its length, *MUC12* has a very interesting mutation pattern that is not readily apparent along with a few interesting locations. A wide range of mutations in *MUC12* have been found to associate with many BRCA histological subtypes as well as UEC. Most striking is estrogen-receptor and progesterone-receptor-positive BRCA - stage II (*n* = 279) with 12.2% patient tumors having amino acid altering mutations. Furthermore, UEC has a high mutation rate with 13.5% in stage I (*n* = 149) and 31.0% (*n* = 29) in stage III. Most strikingly, multiple mutations appear to target arginine (R) in BRCA, where it is converted into either cysteine (C) or histidine (H). This is exemplified with the four BRCA mutations occurring at R1220, in which three arginines change to histidines, while the remaining one becomes a cysteine. Another event is seen at R2777 in BRCA, in which two mutations result in histidine and one becomes cysteine. Lastly, three A1933 frame shift insertions and three P4621T mutations and were observed in BRCA. Despite the low mutation rate, this suggests a possible connection between *MUC12* and BRCA, as the mutations appear low in most tissues except for BRCA and uterine corpus endometrial carcinoma (UCEC).

*MUC16* is a very large transmembrane protein whose mutation rate is relatively high in colon adenocarcinoma (COAD) and colon mucinous adenocarcinoma (COMA) cancers, LUAD, bladder urothelial carcinoma (BLCA), PDAC, and UEC (Figure 1E). Non-damaging nonsynonymous mutations occur in 62.5% of stage II COAD, which is 3.3-fold higher than silent mutations. Stage II COMA has 13 amino acid alerting mutations in 8 specimens, a mutation rate of 1.63 mutations per patient, which is 2.2-fold higher than the silent mutation rate. Stage I READ has twice as many samples and sees a similar mutational rate of 1.63 mutations per patient. LUAD shows a high but roughly flat amino acid damaging mutation rates of 67.7%, 73.5%, 72.9%, and 66.7% across stages I–IV, respectively. LUAD shows a trend of increased nonsense mutations in *MUC16*. UEC shows a high degree of nonsense mutations as well. In the case of PDAC, 43.0% of specimens have amino acid altering mutations, with 14.9% of these mutations resulting in frameshifts or deletions. Oddly, 7/17 silent mutations seen in PDAC stage II all occur at the same base (chr19: 9090831) in an A > G manner (Supplementary Table 2), which was observed by The Exome Aggregation Consortium on 12/4/2015 to occur in 1/120,714 samples, but only in an A > C manner [20]. In other cancers, R8606 has four amino acid changing mutations (Supplementary Table 2).

*MUC20* mutations do not appear to be common, only three A515 frameshift deletions standout (Supplementary Table 2 and Supplementary Figure 1). *MUC13* also has a very low mutation profile with three R324W SNVs and two S185 amino acid altering mutations predicted to be damaging. The same holds true for *MUC15*, with perhaps two mutations at S91L being of mild interest.

**Figure 3:**
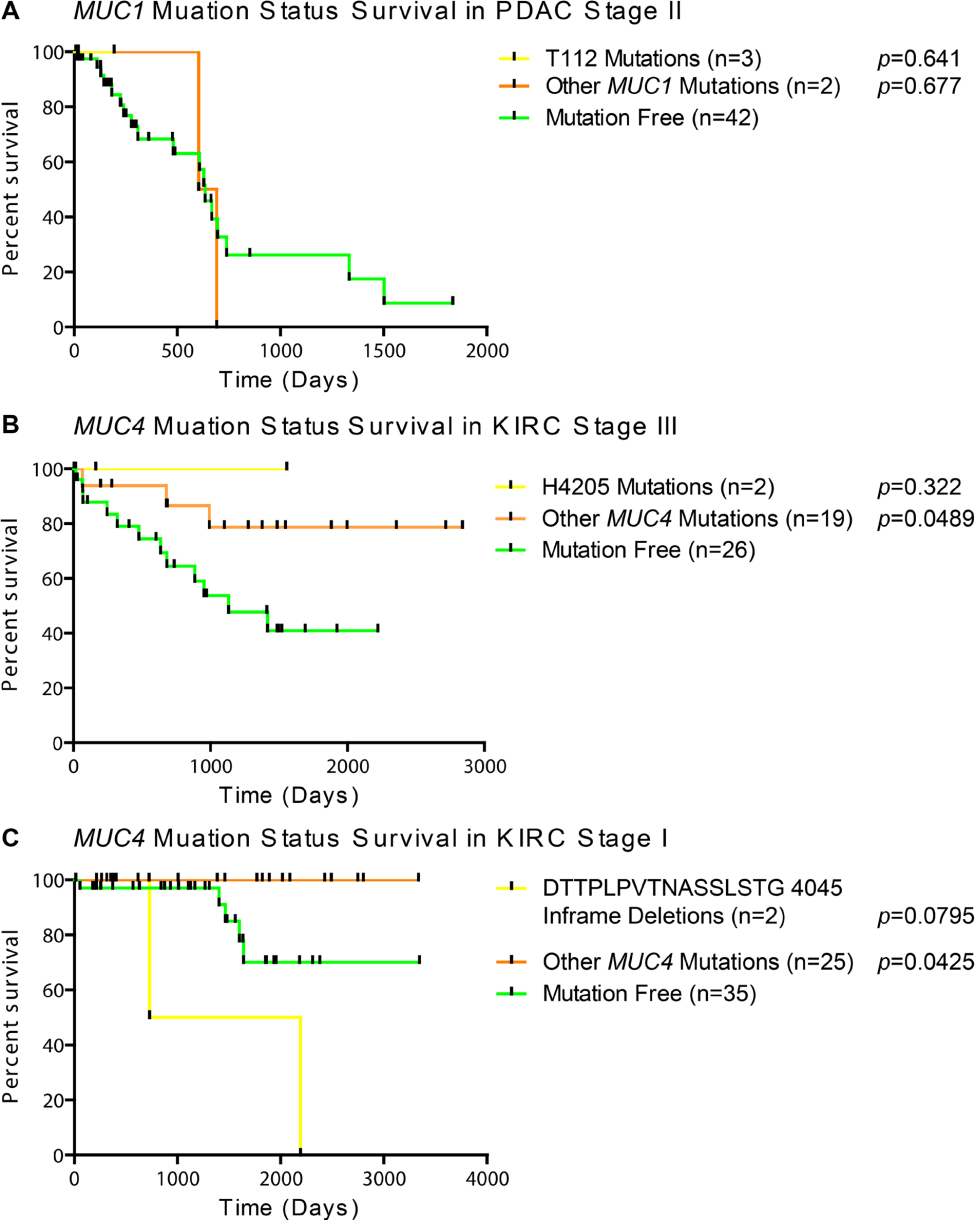
Survival of patients based on mutation status Patients were split into categories based on histological subtype and stage. A minimal cohort size of two was required to examine if the first amino acid change was shared in any other patient(s). (**A**) Kaplan Meier survival plot with Mantel-Cox survival analysis in PDAC stage II patients with no mutations in *MUC1*, only T112P mutations, or any mutations besides T112P. (**B**) Impact of the most common *MUC4* mutations on patient survival by Mantel-Cox analysis in KIRC stage III patients. (**C**) Mantel-Cox survival analysis demonstrates the impact of a repeated inframe mutation in stage I KIRC patients. Yellow lines indicate survival in patients with the specific mutation, orange lines represent patients with a mutation(s) other than the specified mutation, and green lines indicate survival in patients with no mutations in the given gene. Vertical bars indicates a censorship, due to a living or withdrawn patient. All p-values are from a Mantel-Cox survival analysis comparing the adjacent group to the mutation-free group.

Frequent mutations of interest were casually examined for possible impact on survival for when there was more than one mutation residing in the same stage within the same histology. Since half of the MUC1 mutations in stage II PDAC specimens had mutations at T112P in MUC1 (Figure 2A), we examined the mutational impact on survival in the cohort. Unfortunately, examining T112P survival shows that the patients with these mutations have not been enrolled long enough in the TCGA program to generate meaningful statistics, as all three patients had early-censored events (Figure 3A). The observation of a very common mutation at H4205Q in *MUC4* (Figure 2C) caused us to further examine the impact of *MUC4* mutations on patient survival. Despite high occurrences, these mutations appear to improve survival of the patient in stage III KIRC (Figure 3B). A significant change was not observed in stage I KIRC. Further examination of the impact of *MUC4* mutations on patient survival highlights that not all mutations in a gene are potentially beneficial to the patient. A nearly significant (*p* = 0.0795) in-frame mutation at 4045 is associated with increased aggressive behavior of the tumor, while all other mutations appeared to have improved survival compared to patients without *MUC4* mutations in KIRC stage I patients (Figure 3C).

### Mucin mRNA expression in cancer

**Figure 4:**
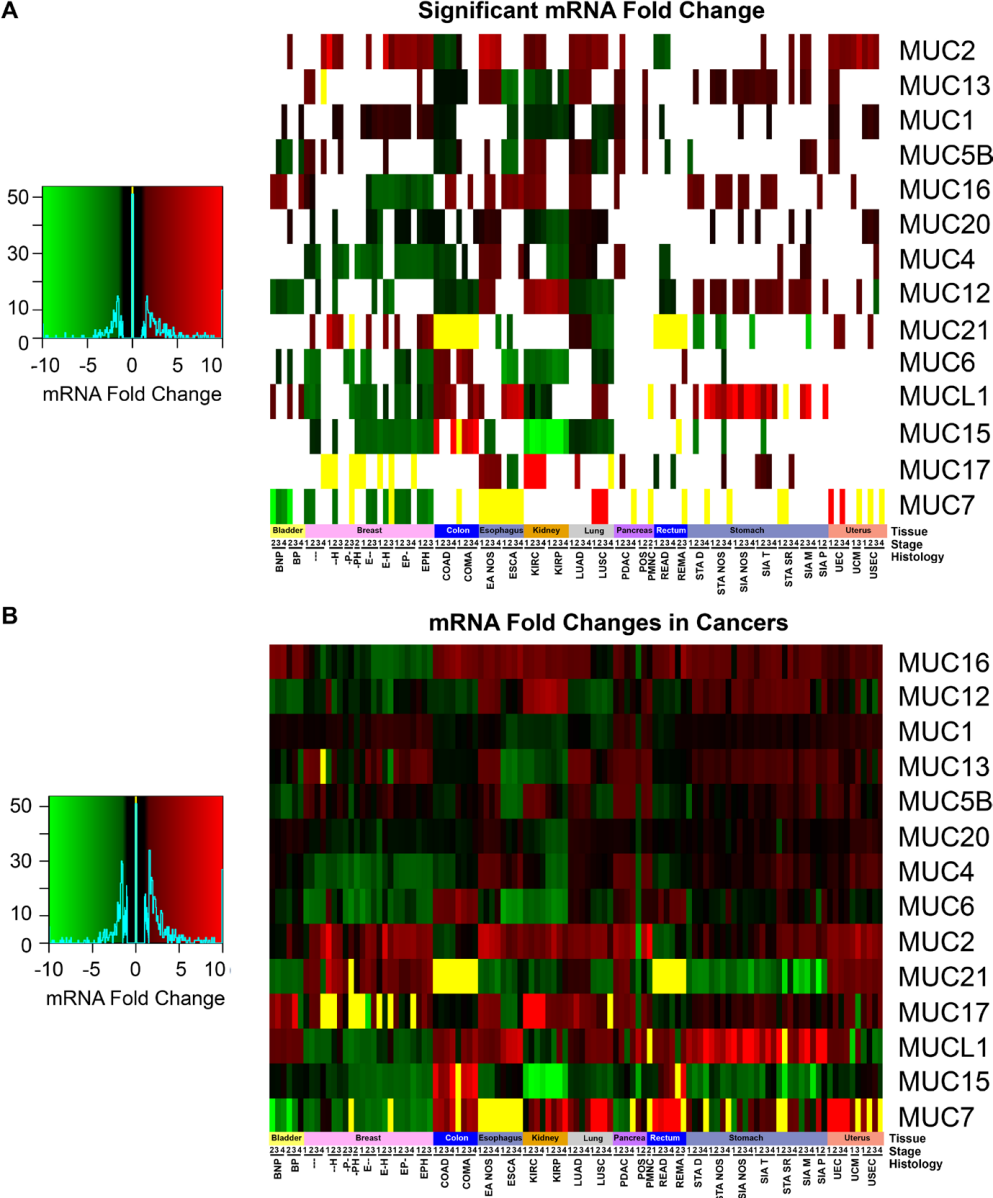
Mucin mRNA expression in cancer mRNA cohorts were separated based on histology and stage, in which a minimum size of two was taken. Supplementary Table 1 contains the full cohort names and sample sizes. Fold change of mRNA in cancer was compared in relation to adjacent non-cancerous normal tissues. If either all the cancer or normal tissue had zero expression, the fold change was set to zero and colored yellow. (**A**) Cohorts which had a significant fold change as determined by a Mann-Whitney (*p* < 0.05) were colored. Cases where Mann-Whitney testing would be impractical had the yellow bars appear in the heatmap. All other cases (*p* > 0.05) have white where the fold change is insignificant statistically. (**B**) The fold change between normal tissue and the cancer cohort was displayed regardless of significance. Heatmap color scales are depicted on the left.

Primary solid tumor mRNA expression data were separated by tissue, histology, and stage and were compared to the respective normal non-cancerous tissues. In comparison to the normal tissue, no change in mucin expression was observed to be unilaterally altered in the same direction through all tissues, which highlights the importance of the tissue origin (Figure 4A and 4B). *MUC1* has very high expression compared to the other mucins in cancerous tissues examined, except for a minor decrease in expression in the colorectal cancers (Supplementary Figure 2). *MUCL1* shows high expression in stomach cancer, especially in SIA NOS where up to 16.5-fold changes were observed. Colorectal cancers are the only tissues to display a significant decrease in *MUC2* (Figure 4A). *MUC2* shows an interesting role with esophageal histological subtypes, showing a significant 5.5-7.1 fold increase for esophagus adenocarcinoma not otherwise specified (EA NOS) in comparison to esophagus squamous cell carcinoma (ESCA), where the latter showed no significant change. Only four of the mucins examined showed similar trends in expression changes between ESCA and EA NOS when significant. Like the esophageal histological subtypes, distinct mucin expression profiles were observed in the lung histological subtypes, LUSC and LUAD, as 9/15 mucins show a contrary trend in the significantly altered mRNA profiles. Contrastingly, KIRP and KIRC have very similar mucin expression profiles between the histological subtypes, only disagreeing in the in the regulation of *MUC4* and *MUC17*. We observed a very strong distinction in the expression of *MUC17* between KIRP and KIRC. KIRC shows a dramatic increase in *MUC17* expression, ranging from 15.4 to 29.4 fold change, while *MUC17* expression is not significantly altered in KIRP. Despite *MUC17* appearing to be turned off in multiple breast cancer histological subtypes, several cohorts suffer from weak sample size (Supplementary Table 1). Significant decreases in *MUC6* was observed in ESCA ranging from −3.8 to −5.4 fold, while no significant change was observed in ES NOS. *MUC7* appears to have little to no expression in many cancer tissues (Figure 4). Examining a possible role with altered expression of *MUC7* is further confounded by small sample sizes of different histological subtypes in multiple cancers, including BRCA and stomach cancers cohorts (Supplementary Table 1 and Supplementary Figure 3). A drastic fold change in *MUC7* expression is seen in LUSC, ranging from 10.0 to 15.3-fold chance when significant, but this is attributed to the little-to-no expression of *MUC7* in normal tissue, as only 1/51 normal specimens had detectable expression after normalization. *MUC15* primarily shows a widespread decreased expression in cancers compared to normal, particularly strong in KIRC and KIRP, where the expression change ranges from −6.6 to −15.5-fold (Figure 4). Contrastingly, COAD shows significant *MUC15* expression increase, which ranges from 7.2 to 20.2 fold when significant. KIRC and KIRP also share a slight 3.7 to 5.7-fold increase in *MUC12* expression.

### *De novo* expression and silencing of mucins in cancer

**Figure 5:**
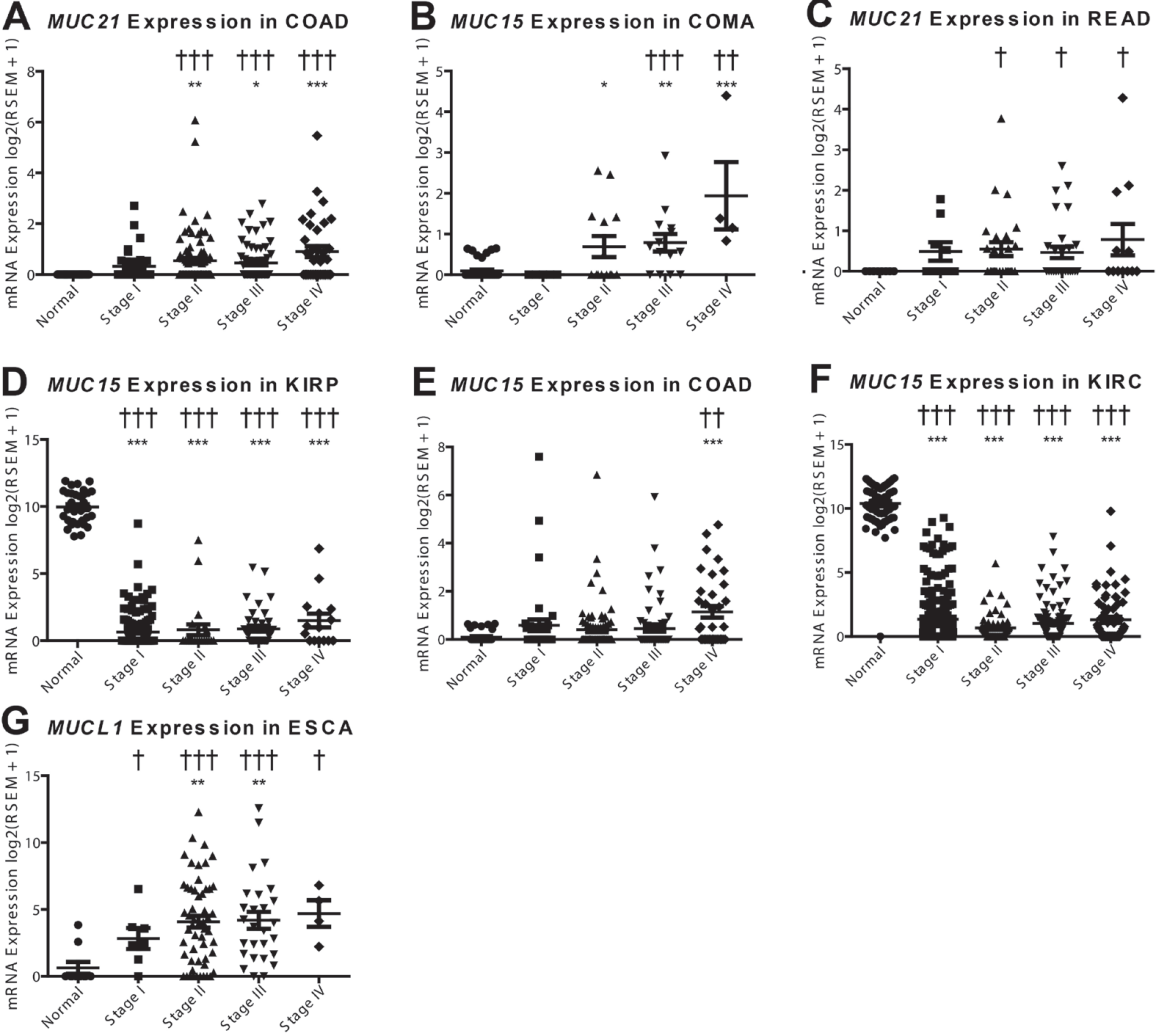
*De novo* expression and silencing of mucins in cancer mRNA expression was plotted for the normal tissue and for each of the four stages in cancer. A Dunn's test in prism was performed for mRNA differences compared to the normal expression (*p < 0.05; **p < 0.01; ***p < 0.001). Supplementary Table 3 Fisher's exact test was consulted for significance (^†^*p* < 0.05; ^††^*p* < 0.01; ^†††^*p* < 0.001) to examine if there is a significant change in the expression status, with expression being on or off. Histological subtypes examined include (**A**) *MUC21* in COAD, (**B**) *MUC21* in READ, (**C**) *MUC15* in COMA, (**D**) *MUC15* in COAD, (**E**) *MUC15* in KIRP, (**F**) *MUC15* in KIRC, and (**G**) *MUCL1* in ESCA.

Mucins are currently being utilized as cancer diagnostic biomarkers; therefore, we sought to explore mucin mRNA profiles for *de novo* expression or silencing in tumors. This endeavor discovered *MUC21* to have significant *de novo* expression, as the normalized expression was not observed in any of the normal colon (*n* = 41) or rectal tissue (*n* = 39), but was seen to increase in COAD and READ (Figure 5A and 5B; Supplementary Table 3). COMA demonstrates an induction of *MUC15* (Figure 5C), as 34 of 41 adjacent non-cancerous samples do not have expression of *MUC15* after normalization. Stage I COMA has zero samples (*n* = 5) with noticeable expression of *MUC15*, while the percentage of stage II-IV patients expressing *MUC15* increased (6 of 14, 10 of 14, and 4 of 4, respectively) and had strong expression ranging from 7.2 to 20.2 fold increase compared to normal adjacent tissue. COAD patients had a relatively modest change in *MUC15* (Figure 5D). Of note, significant impact of mucin mRNA expression changes on survival is seen mainly in the kidney in both univariate and multivariate analyses when corrected for false discovery rate (Supplementary Table 4). Effect of *MUC21* expression increase on survival was significant in both univariate (*q* = 0.005) and multiple regression (*q* = 0.003) showing a hazard ratio (HR) of 1.9 and 2.1 respectively, although only for KIRP, which may signify an underlying harmful role of *MUC21* functioning in cancer.

In contrast to the increased *MUC16* expression in COMA and COAD (Figure 5C and 5D), we observed expression silencing for *MUC15* in kidney histological subtypes KIRP and KIRC (Figure 5E and 5F). There is also a questionable status of *MUC7* expression in BRCA, in which most histological subtypes appear to have some specimens without *MUC7* expression after normalization; however, triple negative breast cancer and low sample size confounds the analysis of *MUC7* in BRCA (Supplementary Table 1 and S3, Supplementary Figure 3). Furthermore, *MUCL1* expression is significantly induced in ESCA; however, a small fraction of normal tissues also express *MUCL1* when normalized (Figure 5G).

### Mucin copy number in cancer

**Figure 6:**
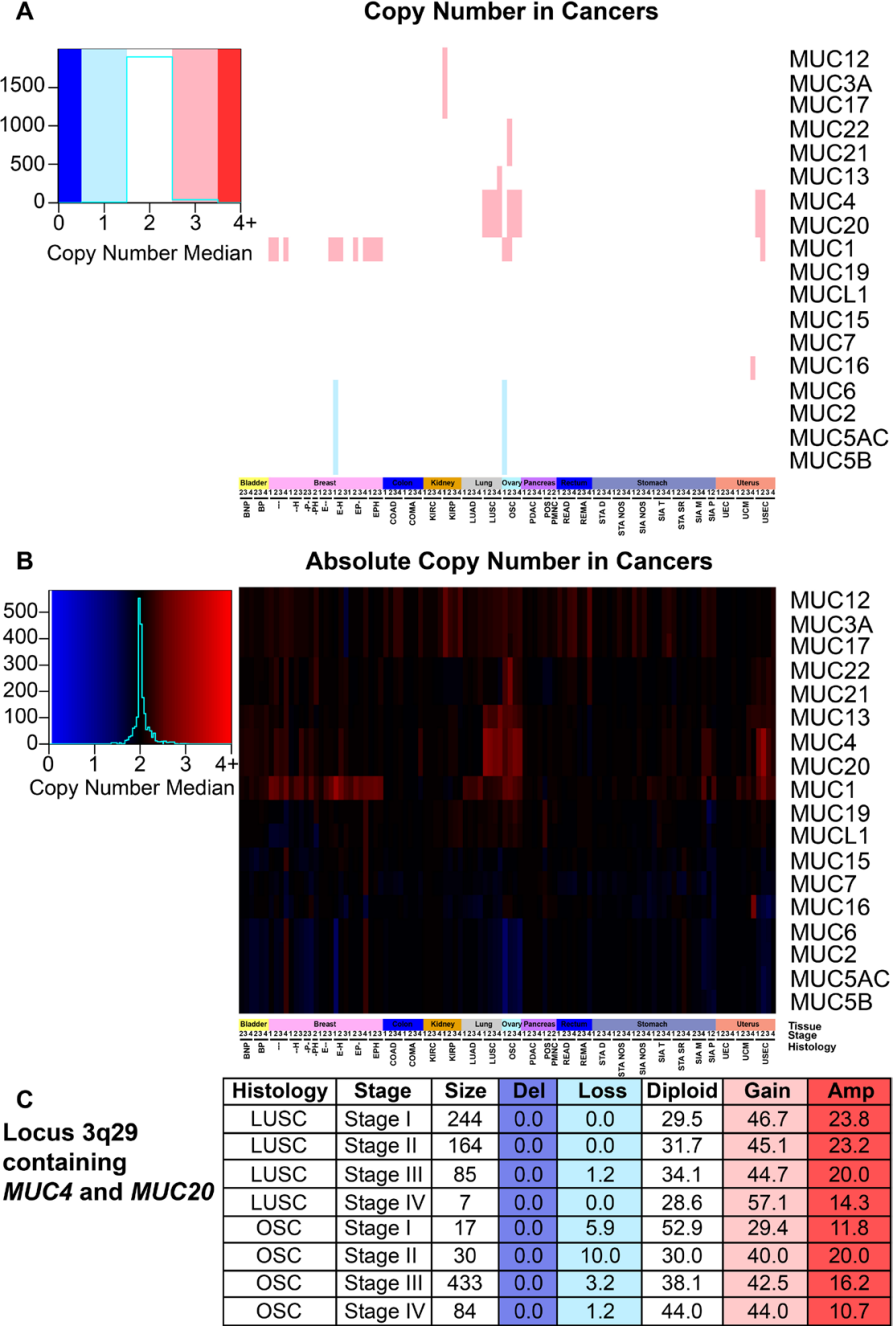
Mucin copy number alterations in cancer Patients were split into cohorts with a minimum size of two for histology and stage. Supplementary Table 1 contains the full cohort names and sample sizes. Figure key color is determined by the median of the copy number, which was determined by the calculated segmean as described in methods. Heatmap color scales are depicted on the left. (**A**) Copy number status that deviated at least 0.5 from diploid were rounded and considered to have copy gain or loss. (**B**) Copy number median is displayed regardless of copy number status. (**C**) A table highlighting the percentage of patients with the corresponding copy number status for the region 3q29 containing *MUC4* and *MUC20* in LUSC and OSC. Histology, stage, and patients in the cohort are listed in the first three columns. The percent of patients in the categories are given with the following copy number statuses: deletion (Del; *n* = 0), copy loss (Loss; *n* = 1), diploid (Diploid; *n* = 2), copy gain (Gain; *n* = 3), and amplified (Amp; *n* = 4 or greater).

Next we assessed somatic copy number alterations (SCNAs) and conservatively examined for SCNA occurrence by evaluating the median copy number. Here we report frequent copy number gains in *MUC1* and the locus 3q29 containing *MUC4* and *MUC20* (Figure 6A and 6B). *MUC1* copy gain state predominates for several cohorts of BRCA, stages I and II of ovarian serous cystadenocarcinoma (OSC), and stage II UEC. The region containing *MUC4* and *MUC20* demonstrated increased copy numbers in over 50% of the patients with any stage of LUSC, stages II-IV OSC (Figure 6C), and stages I and II in USEC (Figure 6A and 6B). Several other cohorts demonstrated somatic copy number amplifications in up to 50% of the patients for the genes *MUC2*, *MUC6*, *MUC5AC*, *MUC5B* in locus 11p15.5; however, due to the conservative nature of the analysis and possible sample size, related cohorts were not seen. After adjusting for multiple hypothesis testing within each histology, only *MUC1* in KIRP was found to significantly impact patient survival for both univariate (HR = 20.1; q = 5.8E-6) and multivariate (HR = 12.9; q = 0.01), indicating a possible negative role of *MUC1* copy number increase in patient survival (Supplementary Table 5).

### Mucin methylation in cancer

**Figure 7:**
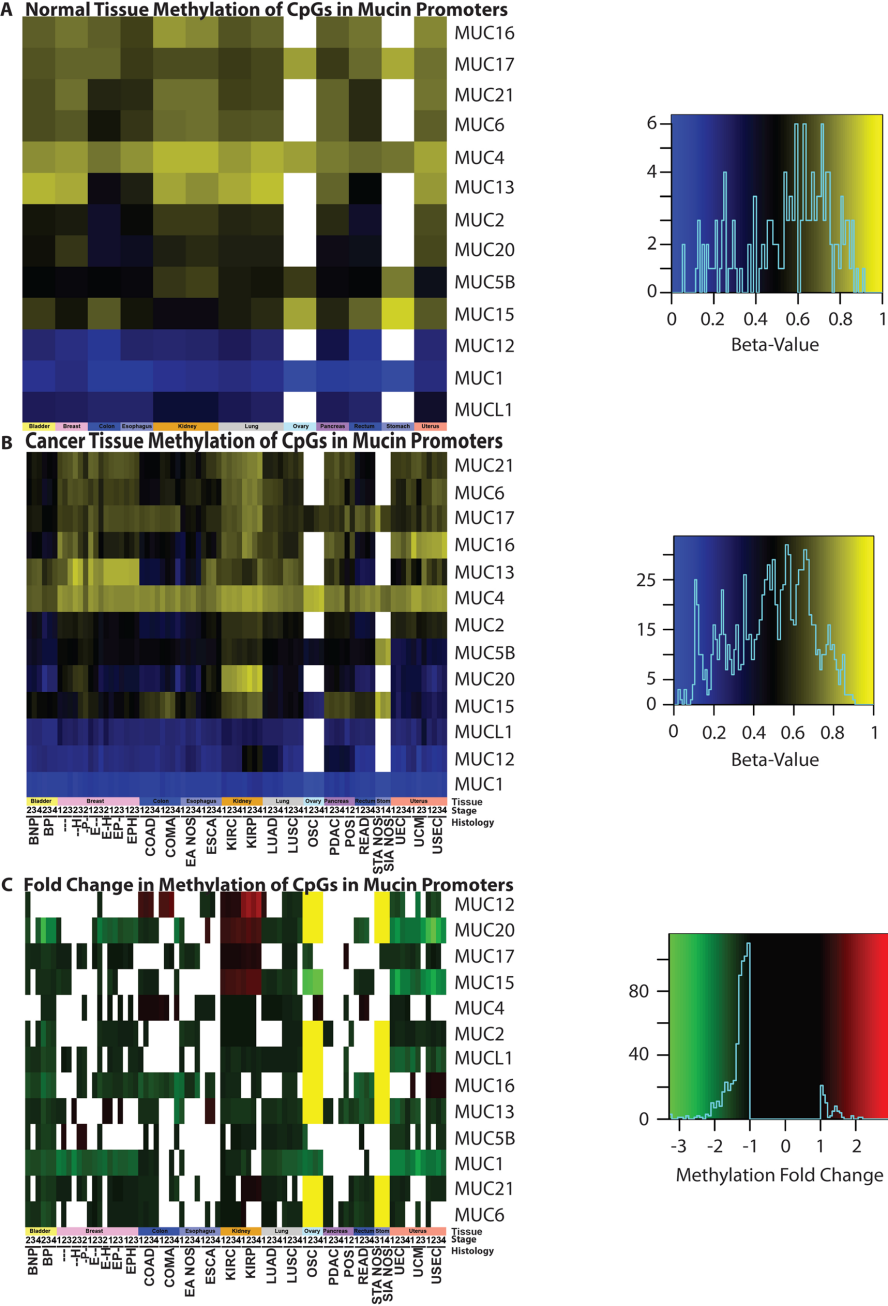
Methylation status of mucins Promoter methylation was examined in (**A**) normal tissue and (**B**) cancer genomes, as well as (**C**) the resulting fold change. Significant (*p* < 0.05) fold changes determined by a Mann-Whitney test are colored, where white indicates a non-significant change between normal and cancer promoters. A yellow bar indicates that a 27K methylation chip was analyzed instead of a 450K chip and the gene was not examined in the chip. A minimum sample size of two was required for each cohort. Supplementary Table 1 contains the full cohort names and sample sizes. Heatmap color scales are depicted on the right.

In the cancers examined, mucin gene promoters typically underwent a significant decrease in methylation (Figure 7). In normal tissues, *MUC1* is the least methylated mucin, except for the normal adjacent tissue in patients with BRCA, where *MUC12* is the least methylated with *MUC1* following behind with 1.1% more methylation (Figure 7A). Following this trend, *MUC12* and *MUCL1* are also lowly methylated in normal tissues. *MUC4* relatively shows high levels of methylation in normal tissue, which is especially evident in cancer (Figure 7B). However, as with the broadly decreased methylation seen in mucins, *MUC4* shows significantly decreased methylation in 34 cancer cohorts and increased methylation in 12 cohorts. With the transformation into a cancer cell, low methylation states are no longer restricted to *MUC1*, *MUCL1*, and *MUC12*. BLCA cohort alone shows a strong decrease in most mucin methylation (Figure 7). *MUC1*, despite low methylation in normal tissues, demonstrates even lower methylation status across multiple cancers.

Many interesting methylation observations exist, however, a few peculiar instances standout. All histological subtypes of UCEC demonstrate a strong decrease in *MUC15* and *MUC20*, in which the average methylation for both of these genes drops to 50% of that of the normal tissues (Figure 7). The *MUC15* methylation status is significantly decreased in OSC, where the fold change ranges from −2.9 to −3.4, compared to the non-cancerous normal ovarian tissues. Both KIRP and KIRC show a unique methylation pattern for mucin gene promoters. An increase in mucin promoter methylation was not commonly observed in cancers, yet in KIRP and KIRC, *MUC15* promoter methylation increased strongly. Gene promoters for *MUC20*, *MUC17*, and even the typically lowly methylated *MUC12* promoter, demonstrate increased methylation in KIRP and KIRC. The *MUC15* promoter methylation has strong impact on patient survival with LUAD; it was discovered to have a significant (*q* = 0.0001) astounding HR of 64.1 when corrected by a multiple regression analysis and a univariate HR of 30.2 (*q* = 0.0017) (Supplementary Table 6).

## DISCUSSION

Only a small subset of mucins have been studied extensively for their roles in cancer biology. Considering the significant roles played by mucins in cancer biology and patient survival, it is imperative to investigate the role of the multiple mucins across cancers. To further understand the genomic significance of mucins, 37 histological subtypes across 12 cancers were examined for mutations, mRNA, copy number, methylation profile, *de novo* expression and silencing, and the impact on survival. Depending on the tissue and the histological subtype, mucins may or may not be exploited in cancer [3, 21]. The results presented here highlight existing as well as new features, which may serve as potential targets in their respective histological subtypes where cancers are suggested to be utilizing mucin function.

*MUC1* has a well-known role in cancer biology [4, 7]. We highlight here that expression of *MUC1* mRNA is higher than most mucins in a majority of cancers and may in part be fostered by increased demethylation of the promoter in multiple cancer tissues (Figure 7). *MUC1* is overexpressed in more than 90% of breast carcinomas [4] and we hereby report that copy number might play a significant role in this tissue, as copy gain was frequently seen in breast cancer (Figure 6A and 6B). These observations are further supported by a significant correlation of *MUC1* mRNA in breast cancer with copy number (*q* = 3.65E-09) (Supplementary Table 7), but not with methylation (Supplementary Table 8), after correcting for multiple hypotheses. Furthermore, we report that only a few cancer subtypes carry *MUC1* mutations, where extracellular region point mutation T112P was commonly seen and responsible for 50% of the *MUC1* mutations observed in PDAC (Figure 1 and Supplementary Table 2). The T112P mutation was also observed in other tissues. However, the only cohort with enough mutations to test survival was in stage II PDAC, in which the corresponding patients were too newly enrolled to obtain any meaningful statistics for survival comparisons (Figure 3A).

*MUC2* has a known tumor suppressor role as seen in colorectal cancer where *MUC2* suppresses inflammation [10, 22]. Therefore, the significant decrease in *MUC2* expression in colorectal cohorts is not surprising; however, for the other tissues examined, only increased expression was observed when statistically significant for *MUC2* (Figure 4A). This includes EA NOS, which showed a significant 5.5 to 7.1-fold increase in *MUC2* mRNA. Furthermore, *MUC2* has an increase in mRNA in KIRC, but not KIRP. KIRP is seen to bare a high burden of non-damaging nonsynonymous mutations and increases through the stages up to 50% in stage IV (Figure 1C). Furthermore, *MUC2* is seen to have a Gaussian distribution of mutations around T1538 (Figure 2B), many of which are threonine, a key component for glycosylation [23]. A large part of the Gaussian distribution stem from KIRP, including three T1488P mutations (Supplementary Table 2 and Supplementary Figure 1). These data suggest potential functional significance of a mutationally important domain in *MUC2* for cancer cell aggressiveness. However, it has been demonstrated by immunohistochemical staining that kidney renal carcinoma (*n* = 16) was negative for MUC2 [24], potentially signifying an artifact generated from little-to-no mRNA in normal tissue and a marginal 2-fold change in KIRC (Figure 4). If there is truly low abundance of mRNA, it is rather unclear why mutations in *MUC2* appear to cluster together in KIRP.

*MUC4* is a well-characterized protein for its significance towards cancer biology [9]. Acting as a ligand for the interaction with ErbB, *MUC4* can bind HER2 and activate several downstream signaling proteins, including ERK1/2, Akt, FAK, and c-Src among others [4, 25, 26]. These MUC4-induced pathways play a critical role in cell growth, proliferation, disruption of tight junctions and adherens junctions, tumor progression, and blocking apoptosis [4, 10, 27, 28]. It is no surprise that *MUC4* is overexpressed, associated with poor prognosis, and potentially serves as a biomarker for cancer [4, 28–33]. However, depending on the tissue, decreased expression of *MUC4* can correlate with poor prognosis as well as an improved prognosis, such as the observation has been previously reported in OSC [28, 33]. Therefore, evaluating the presence and the resultant functional significance of *MUC4* genomic alterations in various histological and stage subsets is important to further understand the role of *MUC4* in cancer. Here we also report that although statistically significant changes in mRNA levels are also observed, the fold change is not very drastic (Figure 4A), suggesting that impact on survival may be due to other possible factors, such as post-transcriptional modifications, including altered glycosylation. Furthermore, methylation is known to play a significant role in *MUC4* expression [22]. In normal tissues and in many cancers, the *MUC4* promoter is highly methylated and a moderate decrease in methylation is observed in cancer, except for a very minor increase in OSC and colorectal cancers that does not exceed 1.08-fold increase. Copy number alterations may contribute significantly to *MUC4* expression, as the genomic segment containing *MUC4* and *MUC20* demonstrates copy gains in over 50% of the specimens in all stages of LUSC, later stages of OSC, and in USEC. Positive significant correlations between mRNA and copy number were seen in breast and pancreatic cancer histological subtypes (*r* = 0.14 and *r* = 0.38, respectively) (Supplementary Table 7). Here we also shed light on the significance of mutations in *MUC4*, especially in KIRC. Although *MUC4* is a rather large gene, repeated mutations and matching in-frame deletions in the same position where seen, especially H4205Q, which we believe should be further investigated. Supplementary Table 2 and Supplementary Figure 1 show additional mutations which maybe of functional interest, including *MUC6* and *MUC12*, the latter of which shows a high preference in which base is mutated. It is interesting to note that these genes, *MUC4, MUC6, and MUC12*, were recently identified as being significantly mutated in smokers in contrast to non-smokers [34].

Examining *de novo* expression of mucins to serve as biomarkers led to the finding of *MUC21* in colorectal cancers, although the increase in mRNA is very low. The expression of *MUC7* is observed not to occur in some of the tissues examined and thus added difficultly in interpreting the significance. However, *MUC7* is expressed in normal submucosal glands in the lungs [35]; in which the data presented here shows LUSC with a significant 10.0 to 15.3 fold increase compared to non-cancerous tissue. This high fold change and *de novo* expression of *MUC7* in LUSC is attributed in part due to 50 out of 51 normal adjacent specimens having zero expression after normalization (Figure 4, Supplementary Table 3, and Supplementary Figure 3). Still, the observed increase in *MUC7* expression might lead to a possible novel marker in cancers. Lastly, a possible role of *MUCL1* was explored, which showed an astounding increase in many stomach cancer histological subtypes (Figure 4).

The mucosal profile of the kidney stands out in many areas. Tissue and histology specific mutations are seen to reside in the kidney (Figure 1C and 1D), some of which have a significant impact on survival (Figure 3C and 3D). Furthermore, KIRC and KIRP both show a dramatic decrease in *MUC15* mRNA. MUC15 is an underexplored transmembrane glycoprotein, which has been shown to have various possible roles in cancers [36–38]. In hepatocellular carcinomas, decreased MUC15 expression was seen and associated with more aggressive phenotypes and shorter survival [37]. However, a reverse trend is seen in glioma, where increased MUC15 correlates positively with progression and stage and serves as an independent factor for prognosis [38]. MUC15 also has signaling interactions with key growth-modulating signaling pathways such as the epidermal growth factor receptor and phosphoinositide 3-kinase [36, 37]. Despite the dramatic decrease of *MUC15* in both KIRC and KIRP, *MUC17* saw a 15.4 to 29.4-fold significant increase in only KIRC (Figure 4). KIRP on the other hand, did not show a statistically significant change in *MUC17* expression in all stages, ranging from only 1.9 to 2.4-fold change above normal in all stages. This suggests *MUC17* as a potential biomarker to distinguish between the histological subtypes. The kidney mucin profile is perhaps the most interesting in regard to methylation changes. This study reveals demethylations of mucin CpG are very frequent in cancer (Figure 7). However, *MUC12*, *MUC15*, *MUC17*, and *MUC20* had a significant increase in methylation in only KIRP and KIRC, which goes against the overall observed demethylation of mucins in the cancers examined here. Of these four mucins, only *MUC15* was observed to have a dramatic decrease in KIRP and KIRC mRNA ranging from −6.6 to −15.5 fold change compared to normal expression (Figure 4). Lastly, *MUC15* and *MUC20* methylation also appears to be of interest beyond renal carcinomas (Figure 7). A significant decrease in *MUC15* methylation was observed in OSC and to a lesser extent, *MUC15* and *MUC20* in UCEC. Despite the lack of normal ovarian samples preventing an analysis on the mRNA fold change, *MUC15* and *MUC20* showed significant correlation with mRNA expression and methylation in both ovarian and uterus corpus cancers (Supplementary Table 8). Only *MUC20* showed marginal significance for mRNA upregulation in USEC (Figure 4). Lastly, despite not being of kidney origin, LUAD *MUC15* methylation was associated with a multiple regression HR of 64.1 (*q* = 0.0001) and a univariate HR of 30.2 (*q* = 0.0017) (Supplementary Table 6). Within the kidney, *MUC21* mRNA expression demonstrated significant impact on survival in univariate analysis in KIRP. Furthermore, within KIRP, *MUC1* copy number had a large impact on survival for both univariate (HR = 20.1; q = 5.8E-6) and multivariate (HR = 12.9; q = 0.01) analyses (Supplementary Table 5).

Here we have presented genomic evidence spanning multiple tissues for further exploration of mucin function in cancers. Many significantly aberrant mRNA expression levels were observed in conjunction with histological subtypes favoring certain mucin mutations as well as location specific mutations. It is our hope the data supplied here and in the supplementary information will aid further explorations of potentially novel functions of mucin family members. We would like to highlight that many roles of mucins cannot be explained by genomic analysis alone. Many mucins may have aberrant glycosylation, phosphorylation, subcellular localization, and are involved in functions which may act independently of genomic alterations discussed here [3, 4, 9, 10, 39, 40]. We hope the study presented here will open new lines of investigations into the functional role, biomarker functions, and therapeutic agents against mucins in cancer.

## MARTERIALS AND METHODS

### TCGA data retrieval

The data used here are based upon data generated by the TCGA Research Network: http://cancergenome.nih.gov/. TCGA clinical files, mRNA, copy number, and methylation were downloaded using the TCGA Data Matrix on 10/5/15. DNA mutation oncotated files mapped to hg19 coordinates were downloaded from firebrowse.org [41] on 11/16/15.

### Clinical attributes

Tailored regular expressions were formed for each of the cancer's clinical patient files, in which the information was stored in a new master spread sheet for all downstream processes. Patients with unclear histological subtypes or pathologic tumor staging were removed. Breast cancer histology was formed utilizing the IHC positive and negative results. Stages were aggregated based only on their numerical value. Patients with attributes for both last follow up and days to death had days to death utilized instead of last follow up. Smoking status, age, ethnic origins were recorded as well. All data analyses utilized here were performed with Perl5 version 16.3 (www.perl.org) and statistical calculations were performed in R version 3.1.3 (www.www.r-project.org).

### DNA mutation analysis

Utilizing only the primary tumor data of patients with clinical attributes as mentioned above, mutation annotation format (MAF) files had all patients of the same cancer, histology, and stage merged together and tracked by the patient identifier. Duplicates MAF entries, such as the same patient having whole genome and whole exome sequencing information, were unified into a single entry. Mutations were examined for false positives by examining against the reference base(s) as well as all available normal and resequenced tumor tissue before storing the data into merged MAFs and generating Annovar files through perl.

SNVs were annotated by Annovar, version release Mar 22, 2015 [42]. Annovar output was traced back to the original patient. The aggregate MAF file by histology and stage were then extracted for calculating frequencies. When examining the aggregated MAF file, if a nonsynonymous mutation was observed, the damaging status was examined against Annovar's output, which utilized MetaLR prediction to make a damaging prediction [43]. Perl then generated files in formats to be visualized in Microsoft Excel 2013, GraphPad Prism 5 (GraphPad Software Inc., CA, USA), and MutationMapper [44, 45]. TCGA coordinates were used in MutationMapper; however, coordinates in mutation mapper may disagree. Due to this reason, *MUC16* appears truncated and the x-axis of *MUC4* was extended to meet the last amino acid in the TCGA coordinates.

### Mutation survival

Aggregate MAFs, based on the same tumor, histology, and stage, were examined for non-silent mutations, which had at least two patients with mutations occurring at the same spot. For mutations impacting more than one base, only the first 5’ base was examined. Only mutations of interest were then examined by both R survival library and Prism 5 utilized files generated by Perl.

### mRNA, copy number, and methylation status analysis

Only primary tumor data was analyzed and was compared to normal samples in the same TCGA category (e.g., KIRC and KIRP were considered separate). A minimum of two samples was required for a Mann-Whitney test in R for mRNA and copy number and three samples for methylation. Methylation utilized either a 450K methylation chip or a 27K chip for ovarian and stomach cancers, which had poor 450K chip sample size on the date downloaded. In the event the TCGA analyzed a patient's sample for methylation more than once, the vial closest to the first extraction was used. Transcription start and stop sites were obtained from UCSC hg19 table browser and analyzed for the longest 5′ and 3′ coordinates and omitted any transcript coordinates that have not been verified. These gene coordinates were used for determining copy number and methylation locations. Methylation analysis was performed to examine all methylation locations for all bases starting from the transcription start site and up to and including the 5,000th base upstream. Copy number analysis was performed by first splitting data into histological subtypes and stages and then examining probe intensities utilizing equal weights per probe and per distance. Should the probe extend past the gene, the probe's distance was adjusted to meet only the span it covered on the gene. In another words, a segment mean was multiplied by the distance the probe covered and was divided by total distance. This score was multiplied by the number of probes with the segmean score and divided by all possible probes that cover the gene. The total sum of the segmean scores within the promoter area was multiplied by the number of total segments examined. Two to the power of this score was taken and this was multiplied by 2. Should the score deviate at least 0.5 away from 2.0, a score representing two copies, it was considered altered. Gene expression was log2 (x + 1) scaled. Perl generated data to be visualized in Prism as well as R. Heatmaps were constructed using R package gplots. *MUC3A*, *MUC5AC*, *MUC19* and *MUC22* were not included due to the TCGA annotation file not including these genes.

### *De novo* expression and silencing

To examine if the percentage of gene expression turning on/off was significant, a Fisher's exact test with independence was utilized. To maintain independence, the same patient could not have their normal non-cancerous tissue and tumor mRNA analyzed together. Therefore, four categories were made for each histological subtype and stage specific grouping: expression or no expression for both normal and cancer samples. The category size for patients with only cancer examined was first counted, then normal tissues were counted. Our aim was to make as even sample categories as possible. Therefore, if a patient had both normal and cancer data, the patient was retained in the smaller group (normal or cancer) for comparisons. Normal group received the patient if the sample sizes were equal. Patient identifiers were thus sorted into cancer or normal groups in order of alphanumerical sort comparison in Perl. At the very end of group assignment, the mRNA levels were examined to assess if the patient had or did not have expression. These groups were then examined by the Perl module Text::NSP::Measures::2D::Fisher2::twotailed. Graphpad Prism 5 tested if mRNA levels were different using Kruskal-Wallis test and a Dunns post hoc test to prevent the assumption of a Gaussian distribution.

### Statistical analysis

Survival is defined as the time from diagnosis to death on each patient. The log-rank test was used to compare the survival between groups. Both the univariate and multivariate Cox-proportional hazard regressions were used to evaluate the associations between the copy number, methylation, log2 scaled mRNA expression levels with the survival of each patient [46]. The confounding effects of age, cancer stage (stages 1 and 2 vs. stages 3 and 4), and smoking status when available (smoking history vs. no smoking history) was adjusted in all multivariate Cox-proportional hazard regression models. All Cox proportional hazard analyses were restricted to cohorts in which at least five patients experienced death events, and have at least five patients in each of the categories defined based on the cancer stage or smoking status when adjusted in the model. The Benjamini-Hochberg method was used to control the false discovery rate for each site for multiple comparisons [47].

## SUPPLEMENTARY MATERIALS FIGURES AND TABLES























## Abbreviations

BLCA: bladder urothelial carcinoma
BRCA: breast invasive carcinoma
PAAD: pancreatic adenocarcinoma
STAD: stomach adenocarcinoma
BNP: Non-papillary bladder cancer
BP: papillary bladder cancer
---: triple negative breast cancer
--H: HER2-positive breast cancer
-P-: progesterone-receptor-positive breast cancer
E--: estrogen-receptor-positive breast cancer
-PH: progesterone-receptor and HER2-positive breast cancer
E-H: estrogen-receptor and HER2-positive breast cancer
EP-: estrogen-receptor and progesterone-receptor-positive breast cancer
EPH: estrogen-receptor, progesterone-receptor, and HER2-positive breast cancer
COAD: colon adenocarcinoma
COMA: colon mucinous adenocarcinoma
EA NOS: esophagus adenocarcinoma not otherwise specified
ESCA: esophagus squamous cell carcinoma
KIRC: kidney clear cell renal carcinoma
KIRP: kidney papillary renal cell carcinoma
LUAD: lung adenocarcinoma
LUSC: lung squamous cell carcinoma
PDAC: pancreas-adenocarcinoma ductal type
POS: pancreas-adenocarcinoma-other subtype
PMNC: pancreas-colloid (mucinous non-cystic) carcinoma
READ: rectal adenocarcinoma
REMA: rectal mucinous adenocarcinoma
STA D: stomach adenocarcinoma diffuse type
STA NOS: stomach adenocarcinoma not otherwise specified
SIA NOS: stomach intestinal adenocarcinoma not otherwise specified
SIA T: stomach intestinal adenocarcinoma tubular type
STA SR: stomach adenocarcinoma signet ring type
SIA M: stomach intestinal adenocarcinoma mucinous type
SIA P: stomach intestinal adenocarcinoma papillary type
UEC: endometrioid endometrial adenocarcinoma
UCM: mixed serous and endometrioid
USEC: serous endometrial adenocarcinoma
UCEC: uterine corpus endometrial carcinoma
SCNA: somatic copy number alteration
MAF: mutation annotation format; hazard ratio.
