# Supplementary material for: Genomic alterations in mucins across cancers
Source: Oncotarget. 2017 May 17;8(40):67152–68. doi: 10.18632/oncotarget.17934 (PMC5620163; doi:10.18632/oncotarget.17934)
Supplement: Supplementary file 1 [file oncotarget-08-67152-s001.pdf]

Genomic alterations in mucins across cancers

Supplementary Materials

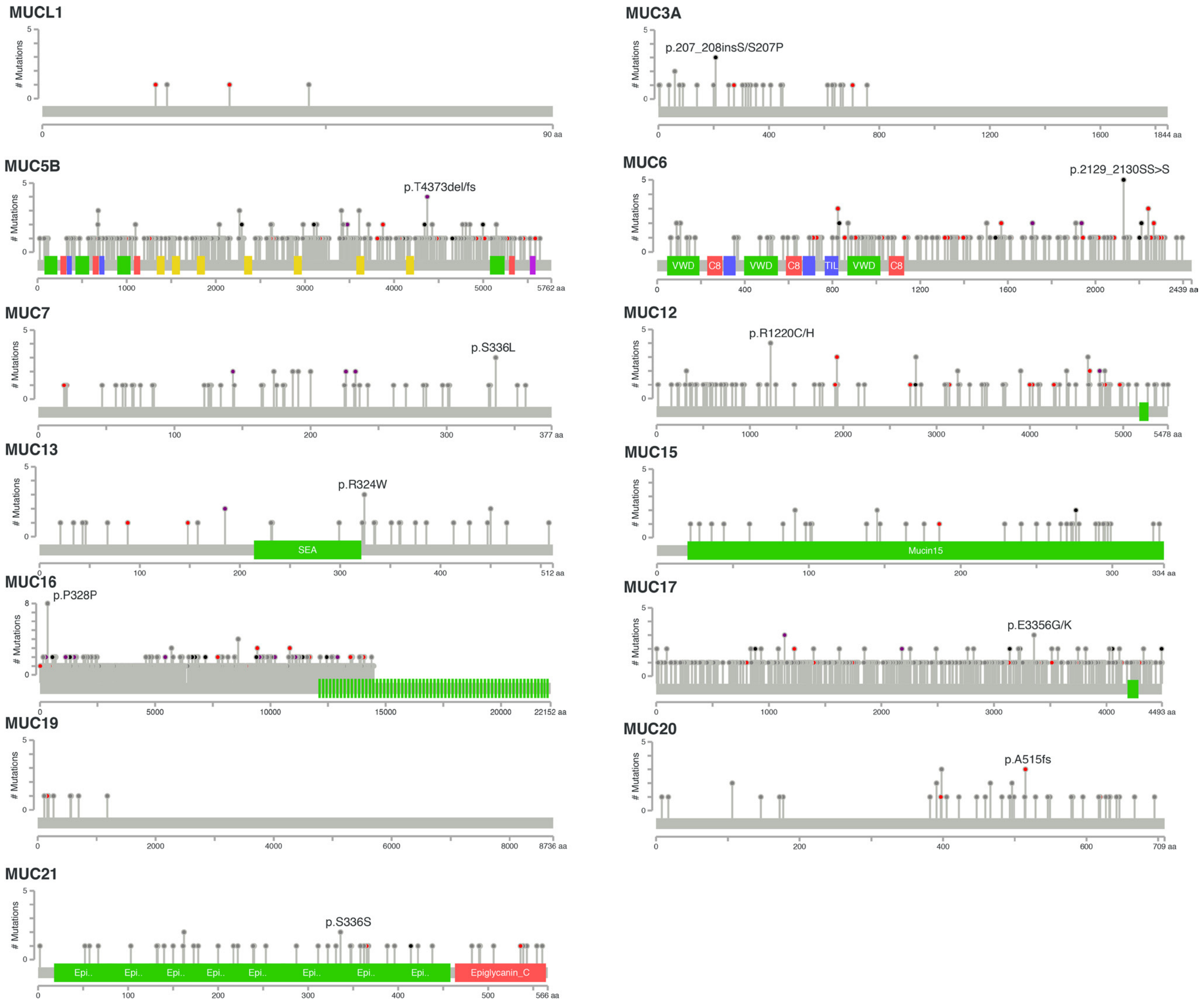

Supplementary Figure 1: DNA mutations by location. DNA mutations of all cohorts were aggregated together to examine for commonly mutated regions. Figures were generated by cBioPortal Mutation Mapper [44, 45], in which each lollipop denotes a unique mutation location. Multiple mutations are separated by a forward slash. Red circles indicate a frameshift, nonsense, or a splice site mutation. Black circles denote inframe additions or deletions. Grey circles indicate either silent or nonsynonymous mutations. Purple indicates multiple color categories reside at the location. Green, red, and yellow bars indicate domains. TCGA coordinates were used; however, if the coordinates in mutation mapper were different, extension is seen, such as MUC12, or an abrupt stop is observed, such as MUC16.

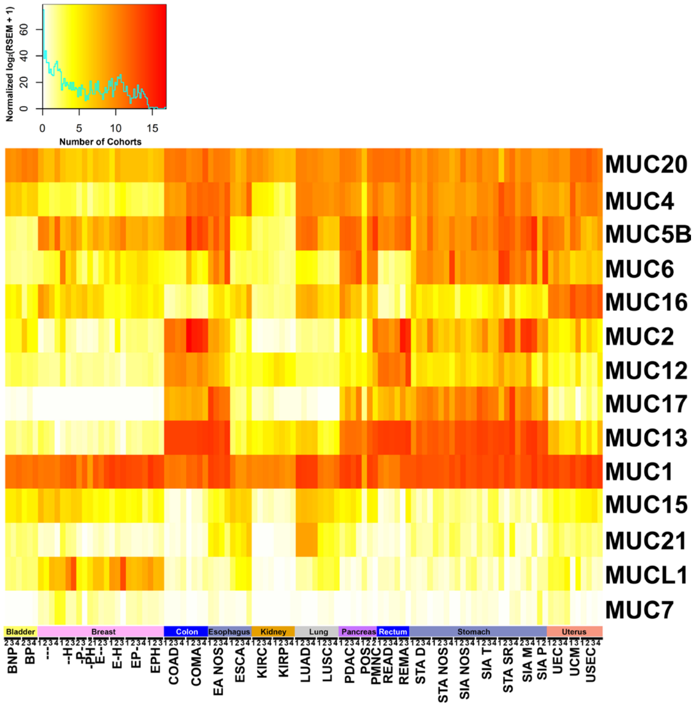

Supplementary Figure 2: Heatmap of mucin mRNA in cancer. mRNA cohorts were separated based on histology and stage, in which a minimum size of two was required. Supplementary Table 1 contains the full cohort names and sample sizes.

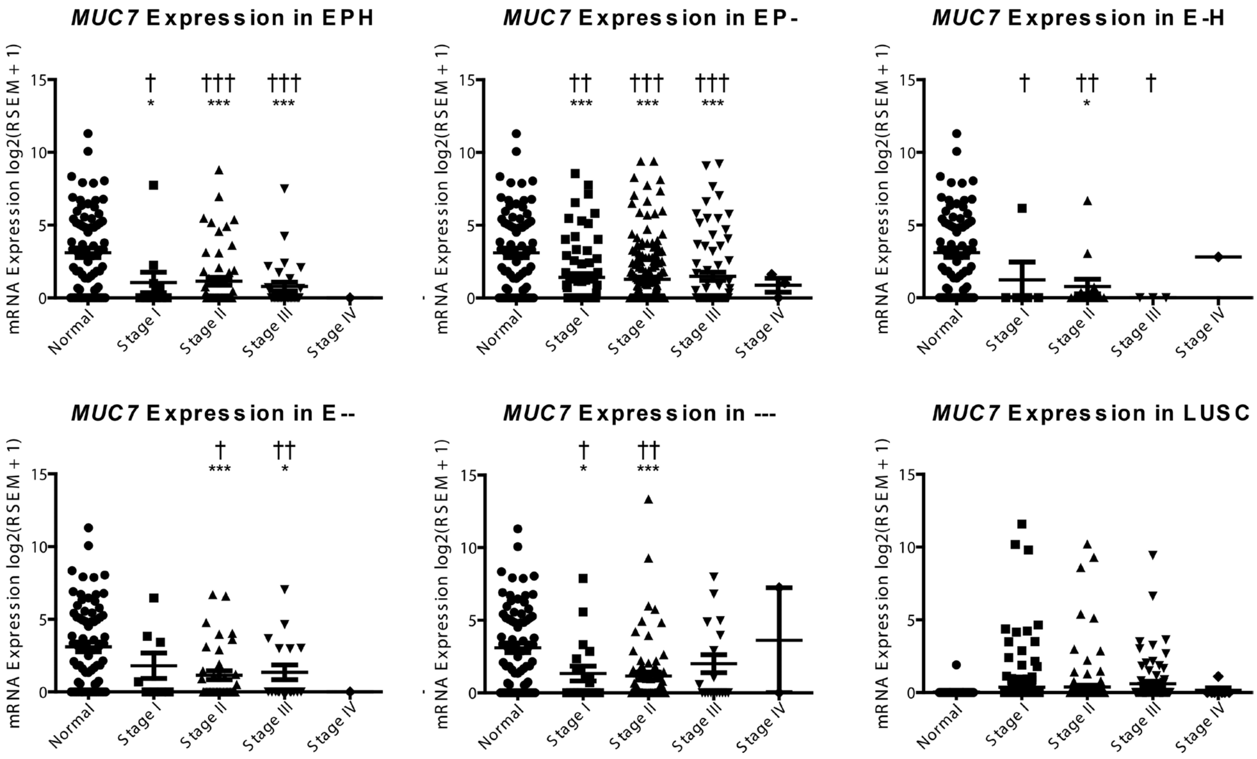

Supplementary Figure 3: *De novo* expression and silencing of *MUC7* examined in breast cancer. mRNA expression was plotted in normal adjacent tissue and each of the four stages in cancer. A Dunn's test was performed to examine for mRNA differences compared to normal expression (*$p < 0.05$; **$p < 0.01$; ***$p < 0.001$). The Fisher's exact test in Supplementary Table 3 was consulted for significance ($^{\dagger}p < 0.05$; $^{\dagger\dagger}p < 0.01$; $^{\dagger\dagger\dagger} < 0.001$) examining normalized RSEM for patients with expression and without expression.

Supplementary Dataset 1: DNA mutations by type. See Supplementary_Dataset 1

Supplementary Dataset 2: Percent DNA mutations per kb in stage III cohorts. See Supplementary_Dataset 2

Supplementary Table 1: Cohort sizes and acronym information. See Supplementary_Table 1

Supplementary Table 2: *De novo* expression and silencing calculations. See Supplementary_Table 2

Supplementary Table 3: DNA mutations and amino acid consequences. See Supplementary_Table 3

Supplementary Table 4: Cox proportional hazard model of mRNA impact on survival. See Supplementary_Table 4

Supplementary Table 5: Cox proportional hazard model of copy number impact on survival. See Supplementary_Table 5

Supplementary Table 6: Cox proportional hazard model of promoter methylation impact on survival. See Supplementary_Table 6

Supplementary Table 7: Correlation of mucin mRNA and copy number status. See Supplementary_Table 7

Supplementary Table 8: Correlation of mucin mRNA and promoter methylation levels. See Supplementary_Table 8
